# Computer-Aided Diagnosis Scheme for Determining Histological Classification of Breast Lesions on Ultrasonographic Images Using Convolutional Neural Network

**DOI:** 10.3390/diagnostics8030048

**Published:** 2018-07-25

**Authors:** Akiyoshi Hizukuri, Ryohei Nakayama

**Affiliations:** Department of Electronic and Computer Engineering, Ritsumeikan University, Kusatsu, Shiga 525-8577, Japan; ryohei@fc.ritsumei.ac.jp

**Keywords:** convolutional neural network, histological classification, computer-aided diagnosis, breast lesion, ultrasonographic image

## Abstract

It can be difficult for clinicians to accurately discriminate among histological classifications of breast lesions on ultrasonographic images. The purpose of this study was to develop a computer-aided diagnosis (CADx) scheme for determining histological classifications of breast lesions using a convolutional neural network (CNN). Our database consisted of 578 breast ultrasonographic images. It included 287 malignant (217 invasive carcinomas and 70 noninvasive carcinomas) and 291 benign lesions (111 cysts and 180 fibroadenomas). In this study, the CNN constructed from four convolutional layers, three batch-normalization layers, four pooling layers, and two fully connected layers was employed for distinguishing between the four different types of histological classifications for lesions. The classification accuracies for histological classifications with our CNN model were 83.9–87.6%, which were substantially higher than those with our previous method (55.7–79.3%) using hand-crafted features and a classifier. The area under the curve with our CNN model was 0.976, whereas that with our previous method was 0.939 (*p* = 0.0001). Our CNN model would be useful in differential diagnoses of breast lesions as a diagnostic aid.

## 1. Introduction

Breast lesions, shown as hypoechoic masses on ultrasonographic images, are important indicators of breast cancer. However, it can be difficult for clinicians to accurately distinguish between benign and malignant lesions—and unusual ones, such as malignant-looking benign lesions or benign-looking malignant lesions, often appear at clinical practice. Some studies have found that a positive predictive value, i.e., the ratio of the number of breast cancers found to the number of biopsies, is rather low [1,2].

To improve the number of positive predictive values found at breast ultrasonographies, many investigators have developed a computer-aided diagnosis (CADx) scheme for distinguishing malignant lesions from benign ones [3,4,5], and in these studies, high sensitivity and specificity for the CADx schemes were shown. At clinical practice, experienced clinicians evaluate not only the likelihood of malignancy, but also the likelihood of histological classification for determining patient managements.

Thus, in our previous study [6] we developed a CADx scheme for determining histological classifications of breast lesions on ultrasonographic images using hand-crafted features and a classifier. Nine objective features were extracted from lesions by considering specific features of images commonly used by clinicians for describing lesions. A multiple-discriminant analysis with the nine features was employed to distinguish among the four different types of histological classifications. We also confirmed the clinicians’ classification performances were substantially improved by using our CADx scheme in an observer study [7]. However, the unsatisfactory classification accuracy of our CADx scheme made it unfit to apply in clinical practice.

Many studies have reported that convolutional neural networks (CNN) have achieved outstanding performance in applying segmentation, detection, and classification of lesions in medical images [8,9,10,11,12,13,14,15,16]. In lesion classification, the CNN which can extract complex multi-level features from input images due to its self-learning ability significantly improved classification accuracy when compared with the use of the conventional methods, as it uses a combination of hand-crafted features and a classifier [16]. Therefore, we considered that classification accuracies of the CADx scheme for histological classifications could be improved by the use of CNN.

In this study, we developed the CADx scheme for determining histological classifications of breast lesions using a CNN. Our CNN model was constructed from four convolutional layers, three batch-normalization layers, four pooling layers, and two fully connected layers. We then evaluated the classification accuracy by applying our CNN model to 578 breast lesions on ultrasonographic images.

## 2. Materials and Methods

The use of the following database was approved by the institutional review board at Mie University Hospital. Informed consent was obtained from all observers, and the database was stripped of all patient identifiers.

### 2.1. Materials

**Figure 1:**
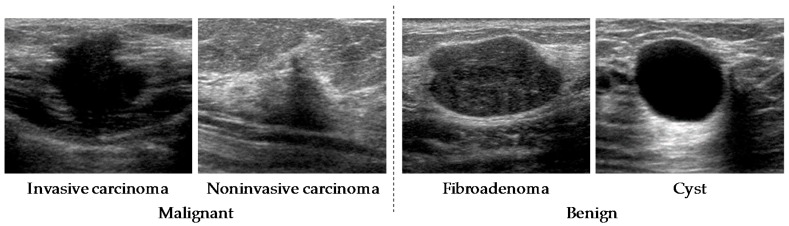
Four lesions with different types of histological classifications.

Our database consisted of 578 breast ultrasonographic images. The lesion sizes in our database were 4–25 mm. These images were obtained from 566 patients using an ultrasound diagnostic system (APLIO™ XG SSA-790A, Toshiba Medical Systems Corp., Otawara, Tochigi Prefecture, Japan) with a 12 MHz linear-array transducer (PLT-1204AT) at Mie University Hospital in 2010. All cases had already been pathologically proven. After the diagnosis of benign cases was confirmed by fine-needle aspiration, the patients were examined again at 6 to 12 months after the initial diagnosis. To avoid the influence of artifacts in the CNN analysis, cases that had undergone a vacuum-assisted needle biopsy, excisional biopsy, or received medication were excluded in this study. The sizes of the images were 716 × 537 pixels with 8-bit grayscale. The database included 287 malignant lesions (217 invasive carcinomas and 70 noninvasive carcinomas) and 291 benign lesions (111 cysts and 180 fibroadenomas). The histological classifications for all lesions were proved by pathologic diagnosis. Regions of interest (ROIs) which included a whole lesion were selected from ultrasonographic images by experienced clinicians. Those ROIs were used for the input of our CNN model. Figure 1 shows an example of lesions with four different types of histological classifications.

### 2.2. Data Augmentation

Although training of the CNN needs a sufficient amount of training data, the number of training data in this study was limited. Because a small number of training data can sometimes diverge learning or cause overfitting in the CNN, we decided to augment the training data by transforming it, using a horizontal flip, parallel translation, and a gamma correction to do so [17].

### 2.3. Architecture of Convolutional Neural Networks Model

**Figure 2:**
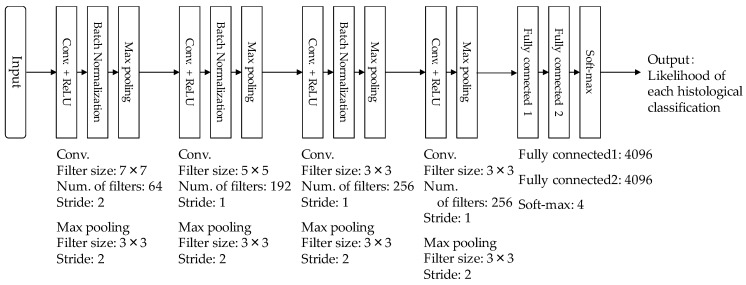
Architecture of our convolutional neural networks (CNN) model.

Figure 2 shows the architecture of our CNN model which was used in this study. Our CNN model was constructed from four convolutional layers, three batch-normalization layers, four pooling layers, and two fully connected layers. Each convolutional layer was followed by a rectified linear unit (ReLU). ROIs with lesions were first resized to 224 × 224 pixels and then given to the input layer of our CNN model. The first convolutional layer generated 64 feature maps with 112 × 112 pixels, using 64 filters with 7 × 7 kernels at stride 2. Once the generated feature maps passed through the normalization layer of the first batch, it then passed the first max pooling layer with a window size of 3 × 3 at stride 2. The second convolutional layer used 192 filters with 5 × 5 kernels at stride 2, and generated 192 feature maps with 55 × 55 pixels. The generated feature maps also passed through the normalization layer of the second batch, then the second max pooling layer with a window size of 3 × 3 at stride 2. Subsequently, the third and fourth convolutional layers with 256 filters of 3 × 3 kernels at stride 2 generated 256 feature maps, with 27 × 27 pixels and 13 × 13 pixels. The batch normalization layer was applied only after the third convolutional layer. The max pooling layers with window sizes of 3 × 3 at stride 2 were employed after both the third and fourth convolutional layers. The generated feature maps from the fourth convolutional layer was merged at two fully connected layers. Finally, the output layer using the softmax function outputted the likelihoods of the four histological classifications (invasive carcinoma, noninvasive carcinoma, fibroadenoma, and cyst).

### 2.4. Training of Convolutional Neural Networks Model

Our CNN model was developed based on the open-source library Keras [18] on Windows 7 Professional (Intel Core i7-6700k processor with RAM 32 GB) and accelerated by a graphic processing unit (NVIDIA GeForce 1070 with 8 GB of memory).

A k-fold cross validation method [19] with *k* = 3 was used for the training and testing of our CNN model. In the validation method, the 566 patients were randomly divided into three groups so that the number of each histological classification was approximately equal in each group (73 patients for invasive carcinoma, 24 patients for noninvasive carcinoma, 60 patients for fibroadenoma, and 37 patients for cyst). One group was used as a test dataset. To assess the possibility of an overfitting of parameters in our CNN model, the remaining two groups were divided into a training dataset and validation dataset of a 90%:10% ratio. This process was repeated three times until every group had been used as test dataset. In this study, the number of ROIs for each histological classification in each training dataset was unified to about 2000 by using data augmentation. Table 1 shows the number of training images before and after augmentation in each dataset.

To select appropriate hyper-parameters, twelve different combinations of hyper-parameters such as the learning rate, mini-batch size, epoch number, and dropout rate were used in our CNN model. We employed the combination of hyper-parameters with the highest classification accuracy in the 12 combinations.

### 2.5. Evaluation of Classification Performance

The classification accuracy of our CNN model was evaluated by using the ensemble average from the testing datasets over the 3-fold cross validation method. The sensitivity [20], specificity [20], positive predictive value (PPV) [20], and negative predictive value (NPV) [20] were defined as:

(1) $$\mathrm{Sensitivity}=\frac{\mathrm{TP}}{\mathrm{TP}+\mathrm{FN}}$$

(2) $$\mathrm{Specificity}=\frac{\mathrm{TN}}{\mathrm{TN}+\mathrm{FP}}$$

(3) $$\mathrm{PPV}=\frac{\mathrm{TP}}{\mathrm{TP}+\mathrm{FP}}$$

(4) $$\mathrm{NPV}=\frac{\mathrm{TN}}{\mathrm{TN}+\mathrm{FN}}$$

Here, TP (true positive) was the number of malignant lesions (invasive and noninvasive carcinomas) correctly identified as positive, whereas TN (true negative) was the number of benign lesions (cysts and fibroadenomas) correctly identified as negative. FP (false positive) was the number of benign lesions incorrectly identified as positive, and FN (false negative) was the number of malignant lesions incorrectly identified as negative. It is noted that the denominators in sensitivity and PPV were coincidentally the same (TP + FN = TP + FP), and the denominators for specificity and NPV (TN + FP = TN + FN) were also the same.

Receiver operating characteristic (ROC) analysis [21] was used for analysis of classification performance. In the ROC analysis, the likelihood of malignancy for each lesion was determined by adding the output values regarding probabilities for invasive and noninvasive carcinomas in a computerized method. We also calculated the area under the curve (AUC) value. The statistical significance of the difference in the AUC value between two computerized methods was tested by using the Dorfman–Berbaum–Metz method [22].

## 3. Results

The classification accuracy was highest when the mini-batch size was 128 ROIs, the learning rate was $10^{-3}$, the max epoch number was 15, and the drop rate was 0.5. Therefore, these hyper-parameters were used in our CNN model.

**Figure 3:**
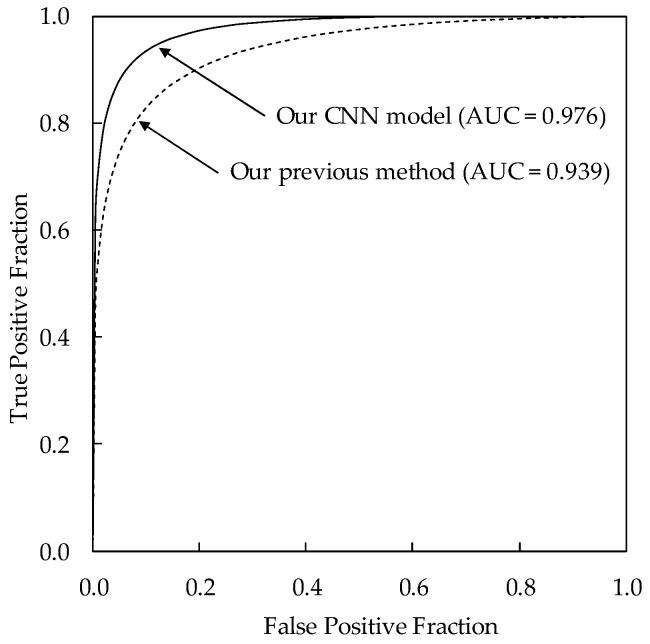
Comparison of the receiver operating characteristic (ROC) curves between our CNN model and our previous method.

Table 2 shows the classification results of the four histological classifications using our CNN model. The classification accuracy of our CNN model was 87.6% (190/217) for invasive carcinomas, 85.7% (60/70) for noninvasive carcinomas, 83.9% (151/180) for fibroadenomas, and 85.6% (95/111) for cysts, respectively. The sensitivity, specificity, positive predictive values (PPV), and negative predictive values (NPV) were 93.0% (267/287), 93.1% (271/291), 93.0% (267/287), and 93.1% (271/291), respectively. The classification accuracies for histological classifications with our CNN model were substantially higher than those with our previous method (71.2% for invasive carcinomas, 55.7% for noninvasive carcinomas, 70.0% for fibroadenomas, and 79.3% for cysts) using hand-crafted features and a classifier [6]. This result indicates the usefulness of CNN in determining histological classifications. Figure 3 shows the comparison between the ROC curves of our CNN model and our previous method. The AUC value of our CNN model was 0.976 (standard error = 0.0052), showing to be greater than that of our previous method (AUC = 0.939, standard error = 0.0094, *p* = 0.0001).

## 4. Discussion

**Figure 4:**
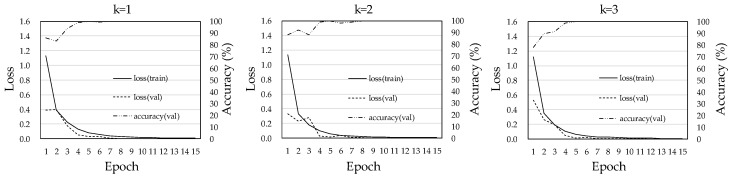
Training curves of our CNN model in each dataset.

Figure 4 shows the training curves of our CNN model for each dataset. The loss curves between the training dataset and the validation dataset tended to similar in the three training phases. Because in the case of overfitting the loss in the validation dataset would be much larger than that for the training dataset [23], we believe that the possibility that there was overfitting in our CNN model was low.

**Figure 5:**
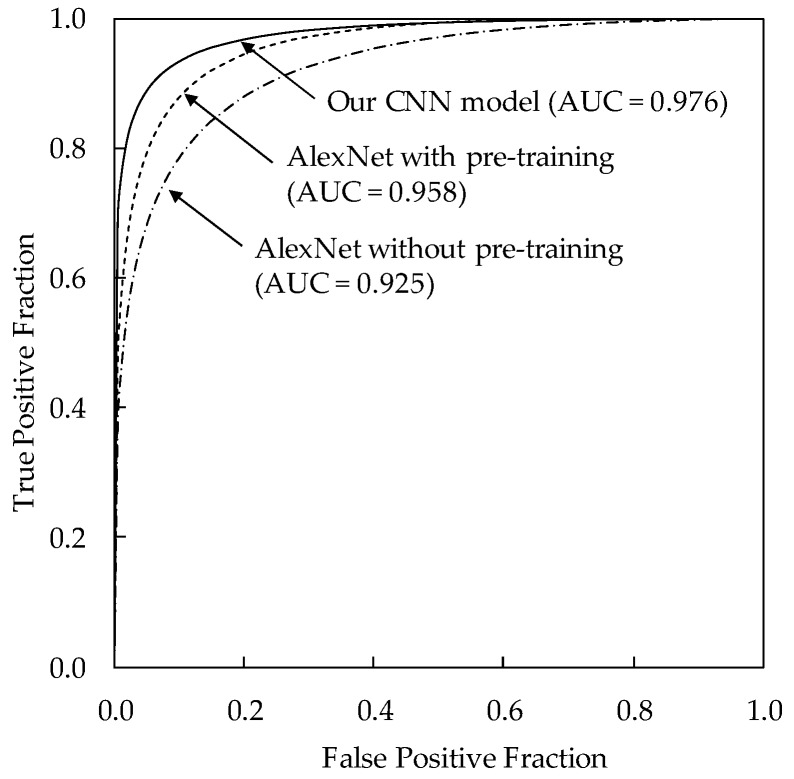
Comparison of the ROC curves for our CNN model, AlexNet without pre-training, and AlexNet with pre-training.

To clarify the usefulness of our CNN architecture, we also evaluated the classification performances for an AlexNet without pre-training [24] and an AlexNet with pre-training [23]. The AlexNet with pre-training had been trained with ImageNet. The hyper-parameters of both AlexNets were given based on a previous study [23]. Both AlexNets consisted of the same architecture and had 5 convolutions, 2 normalizations, and 3 max pooling layers. Each convolution layer was followed by an ReLU [24]. In the last layers, all units were fully connected to output probabilities for 4 classes using the softmax function. The AlexNet without pre-training was trained with our database, whereas the AlexNet with pre-training was fine-tuned for our database. The classification accuracy of the AlexNet without pre-training was 76.5% (166/217) for invasive carcinomas, 64.3% (45/70) for noninvasive carcinomas, 76.7% (138/180) for fibroadenomas, and 76.6% (85/111) for cysts. On the other hand, the classification accuracy of the AlexNet with pre-training was 82.9% (180/217) for invasive carcinomas, 64.3% (45/70) for noninvasive carcinomas, 76.7% (138/180) for fibroadenomas, and 86.5% (96/111) for cysts. The classification performance of the AlexNet with pre-training was higher than that of the AlexNet without pre-training. Because those results were improved substantially by using our CNN architecture, we believe that our CNN architecture was more appropriate for distinguishing between the four different types of histological classifications on breast lesions. The ROC analysis was also employed to compare our CNN model with the AlexNet without pre-training and the AlexNet with pre-training. Figure 5 shows the comparison of the ROC curves for our CNN model, the AlexNet without pre-training, and the AlexNet with pre-training. The AUC for our CNN model was 0.976 (standard error = 0.0052), showing to be greater than both that for the AlexNet without pre-training (AUC = 0.925, standard error = 0.011, *p* < 0.0001) and the AlexNet with pre-training (AUC = 0.958, standard error = 0.007, *p* = 0.0019).

**Figure 6:**
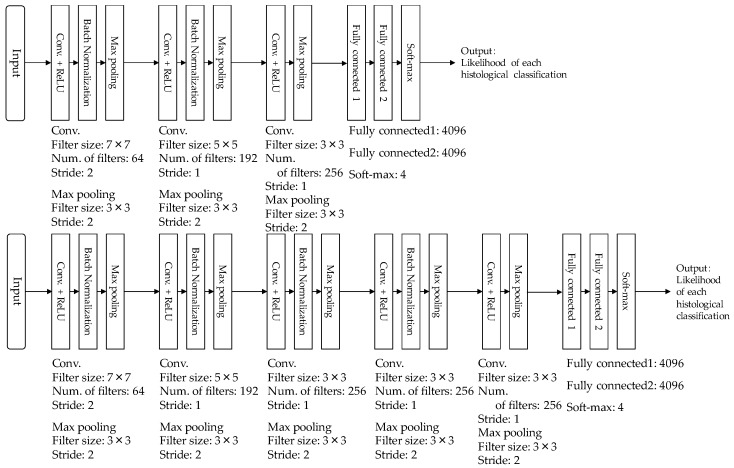
Architecture of two CNN models with three convolutional layers or five convolutional layers.

To investigate the adequacy of the number of convolutional layers used for our CNN model, we compared the classification performances when the number of convolutional layers was changed from 3 to 5. Figure 6 shows the architecture of two different types of CNN models with three or five convolutional layers. Table 3 also shows the classification results when the number of convolutional layers was set to 3, 4, and 5. Our proposed CNN model with four convolutional layers showed the highest classification accuracy among three different CNN models. Although the number of convolutional layers tended to increase in the classification problem, we believed that four convolutional layers was appropriate in determining the histological classification.

To evaluate the validity of the number of filters in each convolutional layer, we compared the classification performances when the number of filters was changed from 0.5 to 1.5 times in our CNN model. Table 4 shows the classification results when the number of filters in each convolutional layer was set to 0.5 times, 1 time, and 1.5 times. The classification accuracy of the CNN model with the combination of the number of filters given in our CADx scheme was higher than those with the other combinations of filters. However, there was no significant change in the classification accuracies due to the combination of the number of filters.

This study has some limitations. One limitation is the that hyper-parameters, such as the number of layers, the number of filters, learning rate, mini-batch size, epoch number, and drop rate in our CNN model, may not have been the best combination for determining histological classifications of breast lesions. Because the number of combinations of hyper-parameters in the CNN is infinite, we evaluated the classification accuracy by using some different combinations of hyper-parameters in our CNN model. The results in this study could be improved by using more optimal combinations of hyper-parameters. We considered that a shallow CNN architecture would achieve a higher classification accuracy than a deep CNN architecture, because the amount of clinical data available for training the model in this study was limited. To clarify the usefulness of the shallow CNN architecture, we compared our CNN with an AlexNet, which is a deep CNN architecture. Although the classification performance for our CNN architecture was greater than that for AlexNet, a deeper CNN architecture might be able to achieve a higher classification accuracy than our proposed one when a larger database is available for training the model in the future. A third limitation is that the size of the ROI was dependent on the size of the breast mass. Because its size and location were given subjectively by experienced clinicians, we can expect there to be some variation among the size and the location of such ROIs, and the classification performance of our CNN model might vary as the ROI is altered. A final limitation is that our CNN model evaluates the likelihood of only four histological classifications, meaning that we would need to make our CNN model applicable to a wider variety of histological classifications in the future.

## 5. Conclusions

In this study, we developed the CADx scheme for determining histological classifications of breast lesions on ultrasonographic images using CNN. Our CNN model was shown to have a high classification accuracy for histological classification, and may be useful in differential diagnoses of breast lesions on ultrasonographic images when used as a diagnostic aid.

## Figures and Tables

**Table 1:** Number of training images before and after augmentation in each dataset.

Pathological Diagnosis	*k* = 1		*k* = 2		*k* = 3	
	Before	After	Before	After	Before	After
Invasive carcinoma	144	2017	145	2030	145	2030
Noninvasive carcinoma	48	2160	46	2070	46	2070
Fibroadenoma	120	2160	120	2160	120	2160
Cyst	74	2220	74	2220	74	2220

**Table 2:** Classification results of four histological classifications.

Pathological Diagnosis	Output of Our CNN Model			
	Invasive Carcinoma	Noninvasive Carcinoma	Fibroadenoma	Cyst
Invasive carcinoma (217)	190 (87.6%)	10 (4.6%)	13 (6.0%)	4 (1.8%)
Noninvasive carcinoma (70)	7 (10.0%)	60 (85.7%)	2 (2.9%)	1 (1.4%)
Fibroadenoma (180)	13 (7.2%)	6 (3.3%)	151 (83.9%)	10 (5.6%)
Cyst (111)	1 (0.9%)	0 (0.0%)	15 (13.5%)	95 (85.6%)

**Table 3:** Comparison of results when the number of convolutional layers was set to 3, 4, and 5.

Pathological Diagnosis	Num. of Convolutional Layers in CNN		
	3	4	5
Invasive carcinoma (217)	189 (87.1%)	190 (87.6%)	195 (89.9%)
Noninvasive carcinoma (70)	58 (82.9%)	60 (85.7%)	51 (72.9%)
Fibroadenoma (180)	143 (79.4%)	151 (83.9%)	151 (83.9%)
Cyst (111)	96 (86.5%)	95 (85.6%)	89 (80.2%)
Total (578)	486 (84.1%)	496 (85.8%)	486 (84.1%)

**Table 4:** Comparison of results when the number of filters in each convolutional layer was set to 0.5 times, 1 time, and 1.5 times.

Pathological Diagnosis	Change Ratio of the Number of Filters in Each Convolutional Layer of Our CNN Model		
	0.5 Times	1.0 Time	1.5 Times
Invasive carcinoma (217)	190 (87.6%)	190 (87.6%)	196 (90.3%)
Noninvasive carcinoma (70)	52 (74.3%)	60 (85.7%)	50 (71.4%)
Fibroadenoma (180)	146 (81.1%)	151 (83.9%)	150 (83.3%)
Cyst (111)	102 (91.9%)	95 (85.6%)	97 (87.4%)
Total (578)	490 (84.8%)	496 (85.8%)	493 (85.3%)
